# Inflammatory processes linked to major depression and schizophrenic disorders and the effects of polypharmacy in psychiatry: evidence from a longitudinal study of 279 patients under therapy

**DOI:** 10.1007/s00406-020-01169-0

**Published:** 2020-07-21

**Authors:** H. H. Stassen, S. Bachmann, R. Bridler, K. Cattapan, D. Herzig, A. Schneeberger, E. Seifritz

**Affiliations:** 1 Institute for Response-Genetics, Department of Psychiatry, Psychotherapy and Psychosomatics, University of Zurich, Psychiatric Hospital, 8032 Zurich, Switzerland; 2 Department of Psychiatry, Psychotherapy, and Psychosomatics, University of Halle, 06112 Halle, Germany; 3 Clienia AG, Psychiatric Hospital, 9573 Littenheid, Switzerland; 4 Sanatorium Kilchberg, 8802 Kilchberg, Switzerland; 5 Department of Psychiatry, Psychotherapy and Psychosomatics, University of Zurich, Psychiatric Hospital, 8032 Zurich, Switzerland

**Keywords:** Polypharmacy, Monotherapy, Antidepressants, Antipsychotics, Efficacy, Side effect profiles, Immunoglobulin M (IgM), Anti-inflammatory response system, Genetics

## Abstract

Over the past 2 decades, polypharmacy has become the de-facto standard of acute treatment in psychiatry where patients with psychiatric disorders receive a multiple medication regimen. There is growing evidence for a potential link between major psychiatric disorders and inflammatory processes. Combining these two aspects aims at avoiding polypharmacy attempts among patients with inflammatory activation through alternative treatment strategies. In this study, we addressed the following questions: (1) to what extent can polypharmacy be explained through the factors “diagnosis”, “previous history”, “severity at baseline”, “age”, “gender”, and “psychiatrist in charge”; (2) what are the differences between polypharmacy and monotherapy regarding efficacy and side effect profiles; and (3) what amount of between-patient variance is explainable by the natural antibody immunoglobulin M (IgM) within each diagnostic group. This naturalistic longitudinal study was comprised of 279 patients under therapy with a clinical diagnosis of depressive (ICD-10: “F3x.x”; *n* = 195) or schizophrenic disorders (ICD-10: “F2x.x”; *n* = 84). The study protocol included (1) assessment of previous history by the SADS Syndrome Check List SSCL-16 (lifetime version); (2) repeated measurements over 5 weeks assessing the time course of improvement by the Hamilton Depression Scale HAM-D and the Positive and Negative Syndrome Scale PANSS, along with medication and unwanted side effects through the Medication and Side Effects Inventory MEDIS; and (3) the collection of blood samples from which DNA and serum were extracted. The association between inflammatory response system and psychiatric disorders was detailed by fitting multi-layer Neural Net (NN) models to the observed data (“supervised learning”). The same approach was used to set up prediction models of side effects. Our data showed that polypharmacy was omnipresent. Yet the various polypharmacy regimens had no advantage over monotherapy: we even found slightly larger baseline score reductions under monotherapy, independent of primary diagnoses and for comparable baseline severities. Most patients experienced unwanted side effects. The close link between side effects and treatment regimen was revealed by a linear model in which the mere number of drugs explained a significant (*p* < 0.001) proportion of the observed variance. As to the inflammatory response system: For the F2 patients, our NN model identified a 22.5% subgroup exhibiting a significant correlation of *r* = 0.746 (*p* = 0.0004) between global schizophrenia scores and IgM levels, along with a correct prediction of response of 94.4%, thus explaining 55.7% of the observed between-patient variance. For the F3 patients, our NN model identified a 19.6% subgroup exhibiting a significant correlation of *r* = 0.644 (*p* = 0.00003) between global depression scores and IgM levels, along a correct prediction of response of 89.6%, thus explaining 41.4% of the observed between-patient variance. Polypharmacy is omnipresent in today’s acute treatment of psychiatric disorders. Given the large proportion of patients with unwanted side effects and the strong correlation between side effects and the number of drugs, polypharmacy approaches are not equally suited for every patient. In terms of efficacy, there are no advantages of polypharmacy over monotherapy. Most notably, our study appears to have cleared the way for the reliable identification of a subgroup of patients for whom the inflammatory response system is a promising target of therapeutic intervention.

**Electronic supplementary material:**

The online version of this article (10.1007/s00406-020-01169-0) contains supplementary material, which is available to authorized users.

## Background

### Treatment of major psychiatric disorders

Depressive disorders and social anxiety are among the main causes for the overall disease burden worldwide and, therefore, a major public health concern. Health surveys suggest that about one in every eight women develops at least one clinical depression during her lifetime, while prevalences in males are only slightly less [1]. Schizophrenic disorders and bipolar illness, on the other hand, each affects about one percent of the general population, causing the loss of the ability to work, to have close relationships, and to have a fulfilling life, thus contributing to the worldwide burden of disability and mortality in a similar way. Together, psychiatric disorders account for 21.2% of years lived with disability worldwide [1]. Available treatments, though effective, are unsatisfactory since treatment options are non-causal, and there is no long-term cure for a considerable proportion of patients (F2: 50–60%; F3: 35–50%) [e.g., [2–4]].

One attempt to overcome the limitations of antidepressant and antipsychotic treatments is “polypharmacy”. In this therapeutic approach, patients are no longer treated with a single medication (“monotherapy”) but receive combinations of antidepressants, antipsychotics, mood stabilizers, anxiolytics, antihistamines, and anticholinergics, among others. Over the past decade, the polypharmacy approach has become the de facto treatment standard in psychiatry. The use of multiple medications can in certain cases be the appropriate and necessary therapeutic option [5]. Today, however, one rarely ever finds patients receiving less than two or more medications. This has given rise to concerns regarding patient safety, efficacy of treatment, and unwanted side effects: the more medications a patient receives, the greater the cumulative toxicity, and the greater the likelihood of adverse events due to interactions between medications which may compromise the desired treatment outcome. And surprisingly, there is little to no empirical data that reproducibly demonstrate the superiority of polypharmacy approaches over monotherapy. Results are for the most part inconclusive [6–10]. The most consistent finding comes from a comprehensive Cochrane study, suggesting that a certain subgroup of F2 patients can apparently benefit from antipsychotic polypharmacy without major negative consequences [11]. But here too we have a number of open questions regarding efficacy and long-term safety, and of how to identify the respective patients.

All this raises principal questions: (1) why is there no causal therapy for affective and schizophrenic disorders? (2) Why do antidepressants and antipsychotics that differ greatly in their biochemical design and primary site of pharmacological action display virtually the same efficacy, so that decisions about drug treatments are primarily based on the drugs’ safety and side effect profiles? (3) Why is it impossible, given current knowledge, to make any predictions of whether or not a particular patient will respond to a particular treatment or will experience a particular side effect pattern?

Our knowledge about the multifactorial etiopathology of major psychiatric disorders and the mechanisms of action of currently available antidepressants and antipsychotics is rather limited. It is indeed impossible to make reliable predictions of whether and when a particular patient will respond to a particular treatment. Similarly, it is indeed impossible to make reliable predictions about the specifics of a particular patient’s side effect profile.

### Etiopathology: genetic component

Family studies suggest that major psychiatric disorders aggregate in families but do not segregate, that is, do not follow simple Mendelian modes of inheritance. Evidence for the involvement of genetic factors in the pathogenesis of schizophrenia originates from studies of monozygotic (mz) and dizygotic (dz) twins reared together [e.g., [12]]. An average 55% of mz pairs are found to be concordant for schizophrenic disorders, compared to only 15% among the dz pairs, so that the question arises as to why mz co-twins reared in the same environment have a 3.7-fold higher risk to both suffer from schizophrenic disorders than do dz co-twins. The answer to this question is “genetics” (or paternal epigenetic factors in mz twins only). Yet twin studies made it also clear that “genetics” can explain only part of the etiopathology as 45% of mz twins remain discordant for schizophrenic disorders over a lifetime even though they share a common genome [12], thus indicating that other exogenous and endogenous factors have significant impacts as well.

### Etiopathology: active immune processes (non-genetic component)

Active immune processes appear to be relevant for the development of major psychiatric disorders in a subgroup of patients as suggested by evidence from recent studies [e.g., [13–17]]. For example, inflammation appears to contribute to the development of depression [18–22], several antidepressants and antipsychotics show anti-inflammatory effects [e.g., [23–26]], and several studies demonstrated microglia activation and progressive brain changes in recent-onset schizophrenia [27, 28]. Therefore, the abnormalities of CNS metabolism observed with depressive or schizophrenic disorders might arise, at least to some extent, because genetically modulated inflammatory reactions damage the microvascular system of the brain. Central to this hypothesis is the patients’ genetically influenced inflammatory response, while the initial triggering of the inflammatory reaction is less important, irrespective of whether being due to an endogenous or exogenous event [29].

Interestingly, the between-subject variation of the “natural” antibody immunoglobulin M (IgM) has been found to possess a strong genetic component [30], while chronically elevated IgM levels typically develop years before the first clinical symptoms of the responsible disease occur as is the case, for example, with rheumatoid arthritis^1^ [31]. Moreover, chronically elevated IgM levels appear to be related to a heritable malfunction in the inflammatory response system as suggested by our study of 599 nuclear families (1868 genotyped subjects) ascertained through index cases with a clinical diagnosis of rheumatoid arthritis [30], and may even be linked to autoimmune diseases in general. The pathogenesis of these latter diseases, however, is insufficiently understood, also because the question of autoantibody appearance prior to inflammation—indicating an antibody-driven inflammatory response—could not yet be answered on the basis of empirical data.

### Twins concordant and discordant for schizophrenia

Within the scope of our EU-funded project “EUTwinsS” Braun et al. [12] addressed the intriguing question of whether or not the differences between mz twins concordant and mz twins discordant for schizophrenic disorders might be linked to the “robustness” of the inflammatory response system. This in the sense that mz co-twins concordant for schizophrenic disorders possess a less “robust” variant of the inflammatory response system that can more easily be triggered by endogenous and exogenous factors than the more “robust” variants of discordant pairs. The authors relied on a multidimensional, quantitative concordance measure that provided a high resolution and differentiation when assessing (1) the resemblance of psychopathology patterns between co-twins, and (2) the between-subject variation of the “natural” antibody immunoglobulin M (IgM).^2^

Based on a sample of 71 twin pairs, the authors found that the variation of within-pair psychopathology concordance among the pairs with at least one schizophrenic co-twin was “explainable” in part by chronically elevated IgM levels (24.5% of variance; *p* = 0.0434), thus supporting the hypothesis that mz twins concordant for schizophrenic disorders possess a less “robust” variant of the inflammatory response system which can more easily be triggered by exogenous factors than the more “robust” variants of discordant pairs.

### Patients suffering from schizophrenic disorders

In a study of 100 patients with a clinical diagnosis of schizophrenic disorders, concordance analyses for the observed syndrome profiles yielded approximately normal distributions with surprisingly robust and virtually identical concordance rates when computing all *n* × (*n* − 1)/2 = 4,950 possible between-subject comparisons of the respective syndrome profiles. The mean values lay around 0.536 ± 0.091 (concordance 53.6%), as long as the key syndromes of schizophrenic disorders were part of the profiles. Most interestingly, the observed between-patient concordances were almost identical with the within-pair concordances reported in the literature for mz twins (55%) [12].

For the sample as a whole entity, no significant correlations between IgM levels and psychopathology syndrome scores could be found. However, it was readily possible by means of Neural Net analyses to “construct” a 20–30% subgroup for which significant correlations showed up between IgM levels on one hand, and the core syndromes of schizophrenic disorders on the other (“schizophrenic thought disorders”, “delusions”, “hallucinations”, “ego consciousness”). A simple IgM cutoff value turned out to be insufficient for this selection. For this subgroup of patients, aberrancies of the inflammatory response system, as quantified through IgM levels, appeared to be linked to the pathogenesis of schizophrenic disorders (*r* = 0.7515/0.8184, *p* < 0.0001) [12].

### Study goals

This “Zurich Polypharmacy Study” was designed to address the following questions: (1) prevalence of polypharmacy in three typical psychiatric hospitals (residential mental health treatment centers); (2) extent to which polypharmacy can be explained through the factors “diagnosis”, “previous history”, “severity at baseline”, “age”, “gender”, and “psychiatrist in charge”; (3) time course of symptomatic improvement in comparison to our previous studies of 2788 patients under monotherapy; (4) prevalence and time course of unwanted side effects under polypharmacy in comparison to monotherapy; and (5) the amount of between-patient variance that is “explainable” by chronically elevated levels of the “natural” antibody immunoglobulin M (IgM).

It would be greatly misleading, however, to expect that aberrancies of the inflammatory response system are involved in the pathogenesis of every patient with a diagnosis of major depression or schizophrenic disorders. Rather, currently available empirical data suggest that no more than 25% of patients with psychotic disorders [12], and no more than an estimated 20% of patients with depressive disorders show signs of such aberrancies. At this point, it is worth noting that low-grade inflammation [32, 33] is neither a necessary nor a sufficient condition for an interrelation between psychiatric disorder and inflammatory response system. Therefore, we relied in this study on multi-layer neural nets and machine learning to determine that initially unknown subgroup of psychiatric patients for whom aberrancies of the inflammatory response system are linked to the pathogenesis of their illness, and for whom the inflammatory response system may be a target for therapeutic intervention.

## Data material

This “naturalistic” longitudinal study is observational and comprised of 279 patients under therapy with a clinical diagnosis of depressive (ICD-10: “F3x.x”; *n* = 195) or schizophrenic disorders (ICD-10: “F2x.x”; *n* = 84). The study protocol included (1) assessments of previous history and overall social functioning through the 63-item SADS Syndrome Check List SSCL-16 and 83-item SADS-Supplement SSCL-SUPP (lifetime versions) [34], (2) repeated measurements over 5 weeks assessing the time course of improvement through the 17/21-item Hamilton Depression Scale HAM-D [35] and the 30-item Positive and Negative Syndrome Scale PANSS [36], (3) repeated measurements over 5 weeks assessing medication and unwanted side effects through the 48-item Medication and Side Effects Inventory MEDIS [37], and (4) the collection of blood samples. The repeated assessments regarding the time course of improvement and unwanted side effects were carried out at weekly intervals plus 2 additional assessments at the 3rd and 10th study day.

The syndrome-oriented instrument SSCL-16 extends the ICD-10 definitions by replacing the yes–no dichotomy of diagnostic schemata by the dimensional quantities “schizophrenic thought disorders”, “delusions”, “hallucinations”, “ego consciousness”, “incongruent affect”, “anergia”, “depressive syndrome”, “manic syndrome”, and “suicide”, while the SSCL-SUPP measures the patients’ overall level of functioning, social relations, affective lability, personality traits, somatization, and consumption behavior.

The 17/21-item HAM-D instrument assesses the severity of depressive disorders by means of a single scale, while the 30-item PANSS instrument assesses the severity of schizophrenic disorders in terms of positive, negative, and general psychopathology scales. The 48-item MEDIS instrument details the following side-effect clusters in a quantitative way: “sleep”, “appetite”, “sexuality”, “gastro-intestinal”, “autonomic”, “neurological”, “cardiovascular”, and “cardiac-respiratory”. A minimum baseline score of at least 15 on the HAM-D17 Scale (primary “F3x.x” diagnoses), or of at least 21 on the PANSS-G Scale^3^ (primary “F2x.x” diagnoses), was required at entry into study after washout. Patients were explicitly excluded if diagnosis was due to an organic background or psychoactive substance abuse. Based on these “naturalistic” criteria, about 70–80% of hospitalized patients were eligible for the study so that results are expected to be for the most part generalizable to psychiatric inpatients in other hospitals. We used all scales for all patients, even though no more than about 25% of F3 patients suffer from significant paranoid symptoms and no more than about 35% of F2 patients suffer from significant depressive symptoms.

## Methods

The patients’ characteristics were modeled using quantitative multi-dimensional profiles of psychopathology, previous history, the time course of improvement under therapy, along with the unwanted side effects caused by therapeutic interventions. The respective data originated from the observer ratings on the basis of the SSCL-16, SSCL-SUPP instruments (previous history), and the HAM-D, PANSS, and MEDIS instruments (response to therapeutic interventions). The raw instrument data were summarized for each individual patient in terms of multidimensional syndrome scores, of which the SSCL-16 lifetime global Depression score (DL: 12 items) and SSCL-16 lifetime global Schizophrenia score (SL: 20 items) were used to quantify the clinical overlap between F2 and F3 patients. We defined this overlap in a heuristic way as follows: SL > 35 and DL > 30 for the F2 patients; and SL > 25 and DL > 35 for the F3 patients. Thus, 32.1% of F2 patients (*n* = 27) and 30.8% of F3 patients (*n* = 60) were allocated to the overlap zone.

**Fig. 1:**
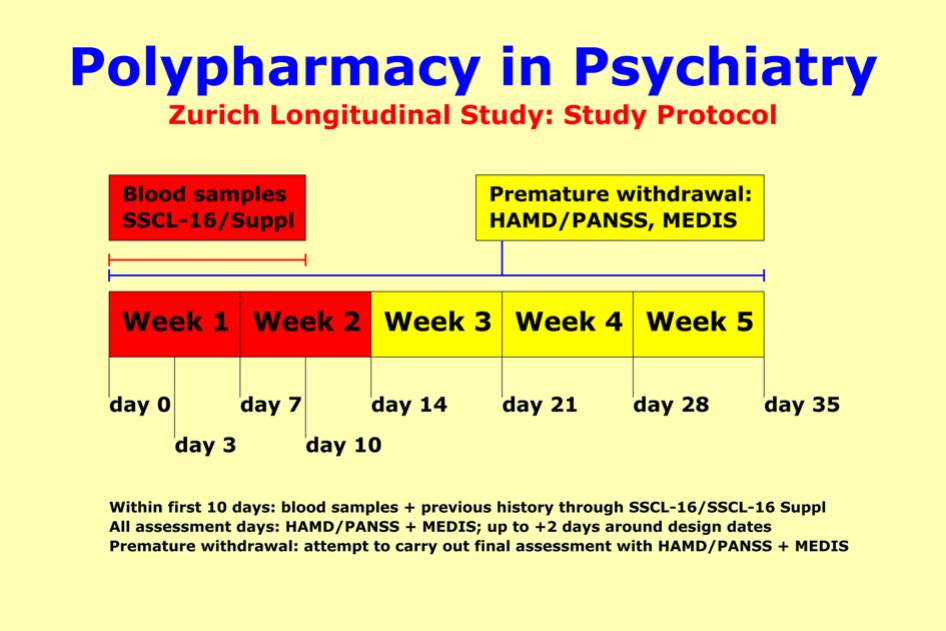
Our study protocol included (1) assessment of previous history and overall social functioning at entry into study; (2) repeated measurements of psychopathology, medication, and unwanted side effects over 5 weeks; and (3) the collection of blood samples

Each individual patient’s response to therapeutic interventions was assessed through a longitudinal profile encompassing up to eight repeated HAM-D, PANSS, and MEDIS scores regarding medications and side effects. The global side effect score *S* was stratified according to the following scheme: (1) no side effects: S ≤ 10; (2) mild side effects: 10 < *S* ≤ 30; (3) moderate side effects: 30 < *S* ≤ 40; (4) severe side effects: 40  < *S* ≤ 50; and (5) very severe side effects: 50 < *S*. The interrelation between treatment regimen and side effects was analyzed by linear regression (Fig. 1).

**Fig. 2:**
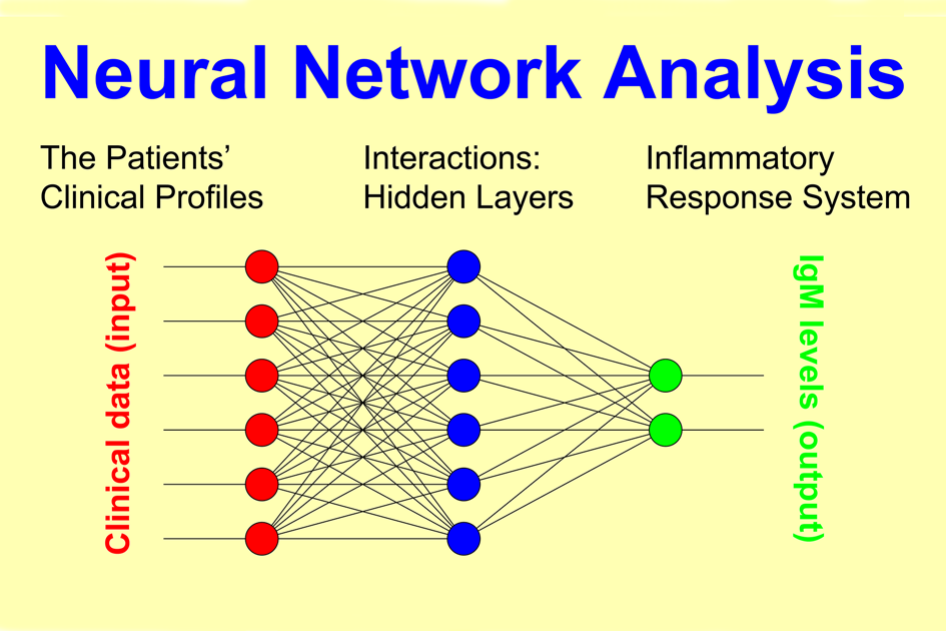
Principal schema of a neural net model where, for example, non-specific IgM levels result from multiple clinical and nonclinical factors connected to each other by complex interactions

We aimed to “learn” the early identification of patients for whom (1) aberrancies of the inflammatory response system may be linked to the pathogenesis of their illness, and (2) the inflammatory response system may be a target for therapeutic intervention. To this end, we iteratively “trained” a multi-layer Neural Net (NN) model as outlined in Fig. 2 (“supervised learning”). The same approach was used to set up prediction models of side effects.

In this methodological approach, “learning” means that for a suitable selection of clinical data—such as previous history or acute psychopathological syndrome scores at entry into the study—a NN model was iteratively determined that predicted, for example, the observed IgM values or treatment response in each individual patient as accurately as possible. Clearly, such a model must not necessarily exist since the hypothesized relationship between the model’s input and output may either not exist in principle, or may not exist uniformly for the sample as a whole entity.

Nonlinear NN models connect the “neurons” of input and output layers via one or more “hidden” layers, thus featuring a relatively large number of free parameters. NN connections are realized through (1) weight matrices and (2) model fitting algorithms minimizing an error function in the weight space (goodness of fit). All outputs are computed using sigmoid thresholding of the scalar product of the corresponding weight and input vectors. Outputs at stage “*s*” are connected to each input of stage “*s* + 1”. The most popular model fitting strategy, the backpropagation algorithm, looks for the minimum of the error function using the method of gradient descent. The basic algorithm is:

**Table:** 

( i)	**Output**:	$s_{i} = \sigma \left[ {\sum\limits_{j}^{{}} {w_{ij} y_{j} } } \right]$	*y*_*j*_ observed	(*i* = *1,2,… N*_i_)
( j)	**Hidden layers**:	$s_{j} = \sigma \left[ {\sum\limits_{k}^{{}} {w_{jk} s_{k} } } \right]$		(*j* = *1,2,… N*_j_)
( k)	**Input**:	$s_{k} = x_{k}$	*x*_*k*_ observed	(*k* = *1,2,… N*_k_)
	**Improvements**:	$\Delta w_{ij} = \alpha \cdot \varepsilon_{i}^{\nu } \cdot s_{j} \cdot s_{i} (1 - s_{i} )$		(*ν* = *1,2,.. p*)
		$$\varepsilon_{i}^{\nu } = y_{i}^{\nu } - s_{i}^{\nu }$$		
		$$\Delta w_{jk} = \alpha \cdot \sum\limits_{i = 1}^{{N_{i} }} {\varepsilon_{i}^{\nu } } \cdot s_{k} \cdot s_{i} (1 - s_{i} ) \cdot w_{ij} \cdot s_{j} (1 - s_{j} )$$		

where *x*_k_ denotes observed stimuli, *y*_j_ observed responses, σ the activation function of sigmoid-type: R → (0,1), *α* the learning rate, and *p* the number of probes (patients). The achievable precision of the model essentially depends on the information included, the quality of underlying data, and the number of intermediate layers implemented to model nonlinear interactions. The computational load, on the other hand, increases exponentially with the number of layers.

Results derived through standard NN approaches, which use 80% of samples for training and the remaining 20% for testing tend to be over-optimistic, in particular in the presence of assessment errors and missing data. By contrast, the *k*-fold cross-validation approach splits the data into *k* roughly equal parts, using *k* − 1 partitions for training, while one partition is used for testing. This process is repeated until each partition has served as a testing set, so that *k* estimates of prediction errors are generated. The resulting prediction errors are approximately unbiased for the “true” error for sufficiently large *k* (*k* ≈ 10 is a typical value in practice). In consequence, we relied on the *k*-fold cross-validation strategy with *k* = 10 throughout the entire project.

## Results

Of the 318 patients recruited within the scope of this longitudinal study 39 (12.3%) dropped out prematurely prior to the envisage study period of at least 3 repeated assessments. Thus, the final study population was comprised of 195 patients hospitalized for depressive disorders (ICD-10: “F3x.x”; 78 males, 117 females; mean age 42.8 ± 12.6 years), and of 84 patients hospitalized for schizophrenic disorders (ICD-10: “F2x.x”; 43 males, 41 females; mean age 38.6 ± 12.2 years). The diagnostic groups did not differ in terms of education (*p* = 0.5161), but showed the expected differences in terms of age distribution and gender composition (gender composition: *p* = 0.0386; age: *p* = 0.0116).

The depressive patients exhibited a mean baseline score of 23.1 ± 5.7 on the HAM-D17 Scale: 51 mild cases (26.2%) with a HAM-D17 baseline score < 20, 68 moderately ill cases (34.9%) with 20 ≤ HAM-D17 baseline score ≤ 24, and 76 severely ill cases (38.9%) with a HAM-D17 baseline score > 24. In terms of HAM-D21 items, 52 patients (26.7%) reported paranoid symptoms, predominantly delusions and hallucinations, and to a much lesser extent, depersonalization and de-realization. The schizophrenic patients, by contrast, exhibited a mean baseline score of 35.8 ± 8.8 on the PANSS-G scale: 16 mild cases (19.3%) with a PANSS-G baseline score < 30, 47 moderately ill cases (56.6%) with 30 ≤ PANSS-G baseline score ≤ 40, and 21 severely ill cases (24.1%) with a PANSS-G baseline score > 40. In terms of the HAM-D17 score, 36 patients (38.3%) reported moderate to severe depressive symptoms.

**Fig. 3:**
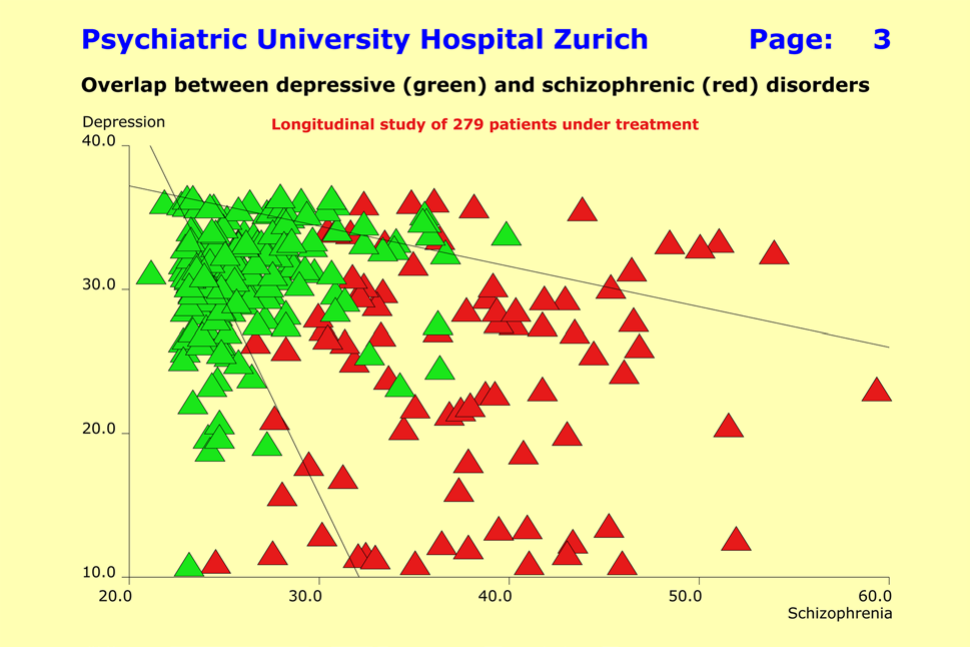
Scatter diagram of patients suffering from depressive disorders (green) and schizophrenic disorders (red) regarding the quantitative syndromes “Depression” (vertical axis) and “Schizophrenia” (horizontal axis). Of particular interest is the heterogeneous clinical picture of patients with functional psychoses and the overlap between clinical diagnoses on the syndrome level

The above symptomatology overlap between clinical diagnoses equally showed up for all psychopathology areas assessed through the study. This well-known fact gave rise to the “continuum hypothesis” in psychiatry many years ago. Using the NN approach, we tested the extent to which patients of overlap zone in the scatter plot of Fig. 3 (where patients were plotted regarding their scores on the global “Depression” and “Schizophrenia” scales) can be identified through IgM levels in combination with other parameters.

The most distinctive symptom complex between depressive and schizophrenic disorders was “Ego Consciousness”, involving symptoms such as depersonalization and de-realization: uncertainty of being oneself, feelings of strangeness or of having changed; delusional belief that one’s appearance, or an organ system, is diseased or changed; feelings of being outside of one’s body; odd or bizarre ideation or magical thinking.

### Polypharmacy

**Fig. 4:**
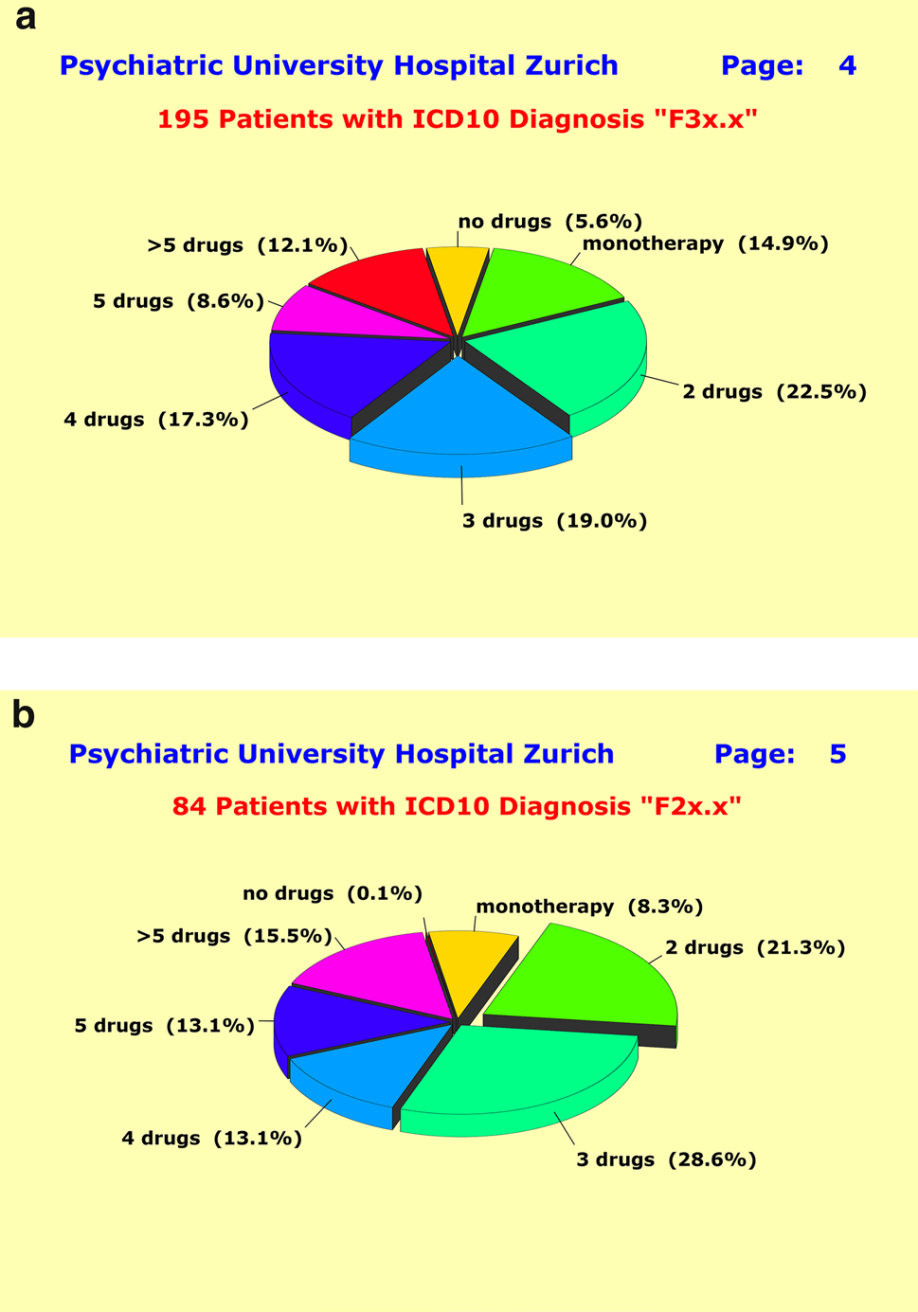
**a** Treatment regimen of 195 patients with ICD10 Diagnosis “F3x.x”. Over the past decade, the polypharmacy approach has become the de facto treatment standard in acute psychiatry, so that one rarely ever finds patients receiving less than two or more medications. **b** Treatment regimen of 84 patients with ICD10 Diagnosis “F2x.x”. Neither clinical diagnosis, previous history, gender and age, nor severity at baseline “explained” the treatment regimens in use. The physician in charge appeared to be the only determinant

Our data showed that polypharmacy was omnipresent. Of the patients with primary F3 diagnoses (“F3 patients”), 5.6% were treated with psychotherapy alone and 14.9% with monotherapy, whereas the vast majority of patients (79.5%) were treated under a polypharmacy regimen (Fig. 4a). This latter subgroup was even larger (91.5%) for patients with primary F2 diagnoses (“F2 patients”)—monotherapy is rarely ever observed (8.3%) among them (Fig. 4b). On average, F3 patients received combinations of 3.2 ± 2.1 and F2 patients of 3.5 ± 1.6 different drugs (medications). There was a non-significant tendency to receive more drugs with increasing age, and a tendency for female patients to receive more drugs at lower doses which reached significance for the F2 patients (*p* = 0.01). Taken together in a linear additive model the parameters “gender”, “age”, and “diagnosis” explained 14.1% of the observed variance in the number of drugs.

The contribution of the parameter “ward” (i.e., psychiatrist in charge) was with 20.4% almost twice as high. Combined together in a nonlinear NN model with interactions, these latter four parameters explained 38.7% of the observed variance which, in turn, implied that more than 60% were just random. The severity of illness and the patients’ suffering had no influence. Even though certain drug combinations were more common in the multitude of observed poly-pharmaceutic approaches, we could not find a clear and generally accepted strategy pursued by polypharmacy.

Defined by a 50% HAM-D17 baseline score reduction and a 40% PANSS-P baseline score reduction,^4^ response to acute treatment was generally modest with response rates of 40.7% (F3 patients) and 30.1% (F2 patients), respectively. The rate of non-responders with no improvement at all throughout the entire observation period was 29.6% (F3 patients) and 44.6% (F2 patients), while partial improvement with baseline score reductions of at least 20% was observed among 29.9% of the F3 and 25.3% of the F2 patients. Most improvement (70%) occurred within the first 2 weeks of treatment.

The various polypharmacy regimens showed no advantage over monotherapy, neither among the F3 patients nor among the F2 patients. Quite the contrary was the case: we found slightly larger baseline score reductions among the patients under monotherapy (and psychotherapy alone) independent of the primary diagnostic group and for comparable baseline severities (Table 1). As the monotherapy subgroups were relatively small, the observed differences did not reach statistical significance.^5^

**Table 1:** The various polypharmacy regimens showed no advantage over monotherapy, neither among the patients with primary F3 diagnoses nor among the patients with primary F2 diagnoses

	Primary F3 diagnoses		Primary F2 diagnoses	
	Polypharmacy	Monotherapy	Polypharmacy	Monotherapy
	*n* = 155	*n* = 40	*n* = 77	*n* = 7
Polypharmacy versus monotherapy				
Severity at baseline	23.4 ± 5.6	22.1 ± 5.6	15.4 ± 6.9	19.0 ± 7.2
Baseline score reduction	−9.1 ± 7.5	−10.0 ± 7.9	−4.3 ± 5.9	−7.1 ± 6.9
Responders	40.6%	40.0%	29.0%	42.9%
Partial responders	29.0%	32.5%	24.9%	28.6%
Non-responders	30.4%	27.5%	46.1%	28.6%

Slightly larger baseline score reductions were found under monotherapy (and psychotherapy alone) independent of primary diagnostic group and for comparable baseline severities. As the monotherapy subgroups were relatively small, none of the observed differences reached statistical significance

**Fig. 5:**
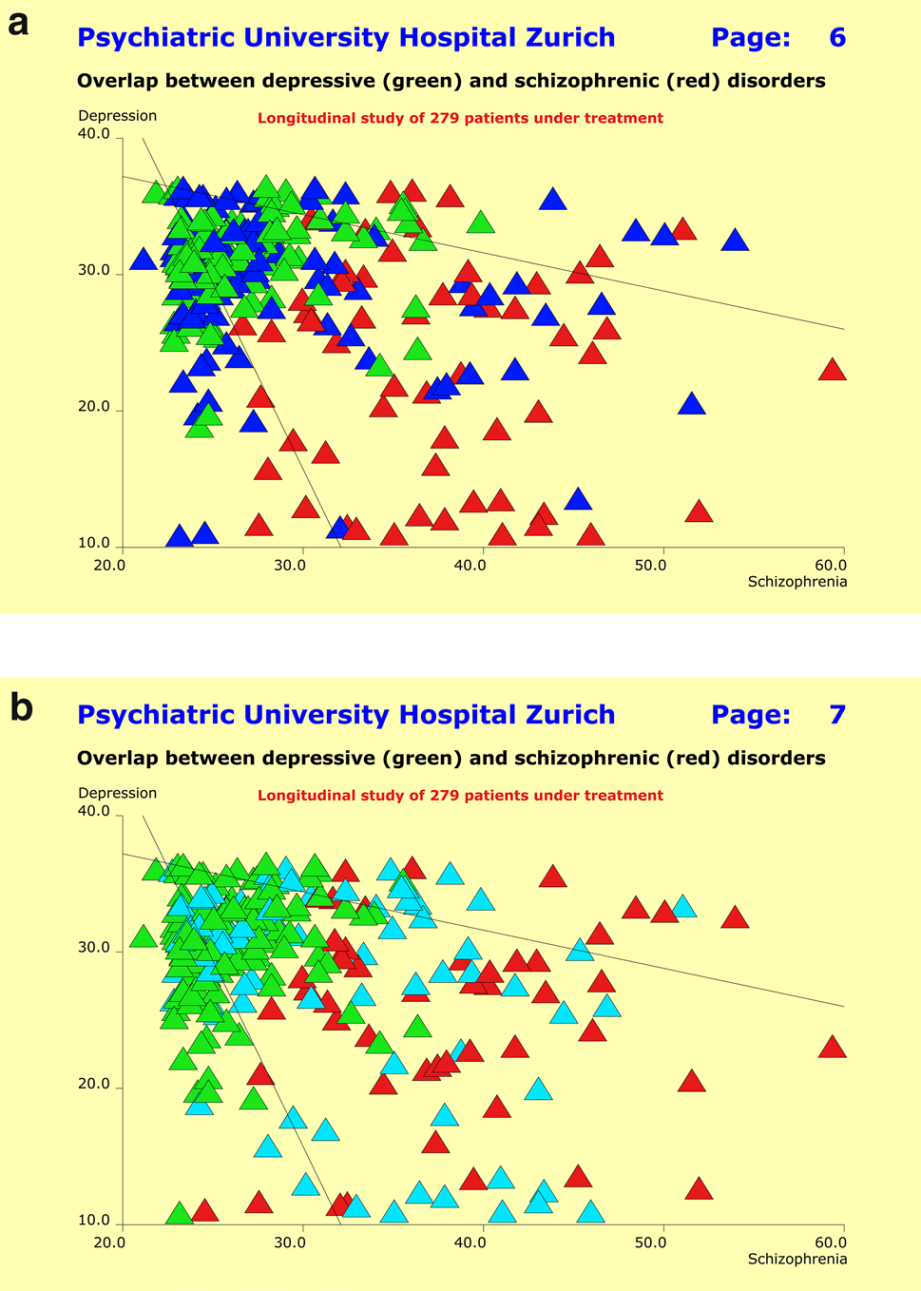
**a** Scatter diagram of patients suffering from depressive disorders (green) and schizophrenic disorders (red) regarding the quantitative syndromes “Depression” (vertical axis) and “Schizophrenia” (horizontal axis). Patients showing a 50% baseline score reduction on the HAM-D17 scale (F3 patients) or a 40% baseline score reduction on the PANSS-P scale (F2 patients) are displayed as dark blue triangles (“responders”) and appear to be randomly distributed over the entire scatter plane. **b** Scatter diagram of patients suffering from depressive disorders (green) and schizophrenic disorders (red) regarding the quantitative syndromes “Depression” (vertical axis) and “Schizophrenia” (horizontal axis). Patients showing absolutely no improvement over the entire observation period (“non-responders”) are displayed as light blue triangles and appear to be randomly distributed over the entire scatter plane

Attempts to establish a linear or nonlinear model that predicts response to treatment or non-response from previous history, the acute state of the disorders at baseline, or the chosen treatment regimens failed altogether. Independent of primary diagnoses, response to treatment appeared to be an autonomic process entirely unrelated to the clinical data collected within the scope of this study. The situation is best illustrated by the scatter of Fig. 3, where responders (Fig. 5a) and non-responders (Fig. 5b) are randomly distributed over the entire scatter plane. Partial responders are unsystematically distributed in a similar way.

### Side effects

**Fig. 6:**
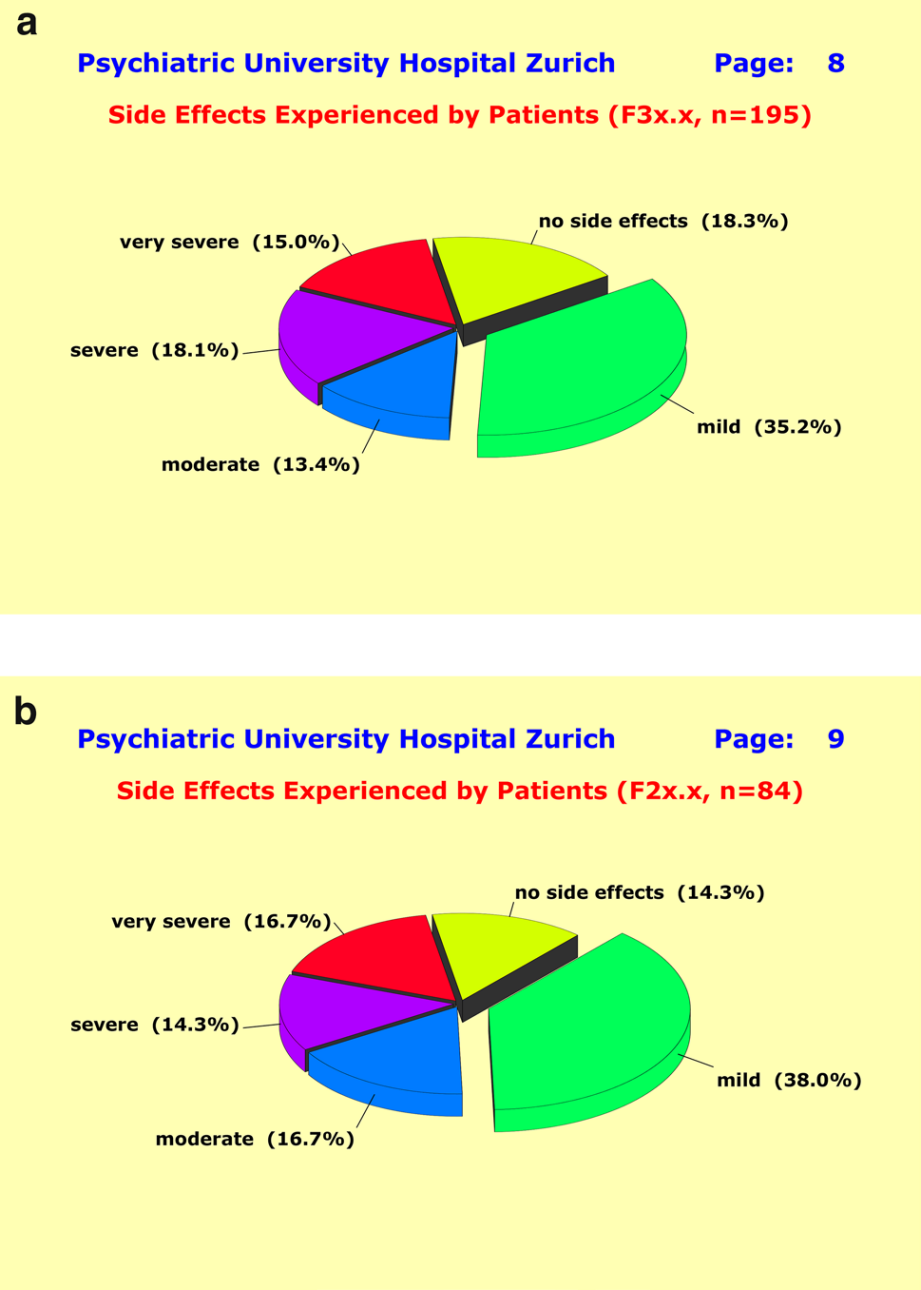
**a** Most F3 patients (81.7%) experienced unwanted side effects in tandem with their treatment regimen (48.6% in mild to moderate, and 33.1% in severe forms). As to response rates, the majority of F3 patients did not benefit from polypharmacy regimen compared to monotherapy. **b** Most F2 patients (85.7%) experienced unwanted side effects in tandem with their treatment regimen (54.7% in mild to moderate, and 31.0% in severe forms). As to response rates, the majority of F2 patients did not benefit from polypharmacy regimen compared to monotherapy

In tandem with the envisaged therapeutic effects, most patients experienced unwanted side effects induced by their psychotropic medications: 84.9% of the F2 (Fig. 6a) and 79.7% of the F3 patients (Fig. 6b). The percentages of the severe forms were 31.0% (F2) and 33.1% (F3), and for the mild to moderate forms 54.7% (F2) and 48.6% (F3). We found a close link between treatment regimen and side effects as revealed by a linear regression model in which the mere number of drugs taken simultaneously explained a highly significant (*p* < 0.001) proportion of the observed variance in side effects irrespective of the substances involved: 17.8% of the variance of sexuality-related side effects, 11.8% of the variance in gastrointestinal disturbances, 13.3% of the variance in cardiac-respiratory disturbances, and 18.4% of the variance in cardiovascular disturbances.

By contrast, when focus was laid on severe side effects, drugs and their interactions with each other came into play as certain drugs can have a much stronger side effect pattern than others. A tentative nonlinear NN model of drugs along with their interactions suggested that some eight substances can explain a major proportion (> 50%) of the observed variance in severe side effect patterns. Given the diversity of polypharmacy treatment regimens in our sample, these results must be regarded as tentative and preliminary, the more so, as the molecular genetic data of this patient sample are not yet available. Once the molecular-genetic data can be included in our side effect models (e.g., cytochrome P450 enzymes) it can be expect that NN-based models can predict the risk of severe side effect profiles in the individual patient with sufficient reliability.

### Inflammatory response system

To explore the extent to which inflammatory processes may be involved in the etiopathology of major psychiatric disorders, we analyzed the “natural” antibody immunoglobulin M (IgM) and determined the amount of between-patient variance that is “explainable” by chronically elevated IgM levels. For the sample as a whole entity, our tentative analyses did not reveal any substantial association between clinical picture and treatment outcome on one hand, and IgM levels on the other. Neither for the parameters “diagnosis”, “previous history”, “severity at baseline”, “age”, “gender”, and “unwanted side effects” nor for the subgroups of treatment responders or non-responders. However, the scatter plots “global schizophrenia score” and “global depression score” versus IgM levels suggested the existence of subgroups of patients for whom the anti-inflammatory response system may have had influenced the patients’ clinical picture and response to treatment.

Therefore, we relied on the patients’ quantitative previous history syndrome scores along with the current baseline severity at entry into study, and used a nonlinear multi-layer NN model to search for the largest subgroup of patients with (1) a close association between clinical picture and IgM levels, and (2) a minimum false-positive prediction error rate regarding response to treatment. As focus was laid on subgroups of patients, the inclusion of constraints regarding false-negative prediction errors did not make sense—there might be other subgroups as well, entirely unrelated to the inflammatory response system.

**Fig. 7:**
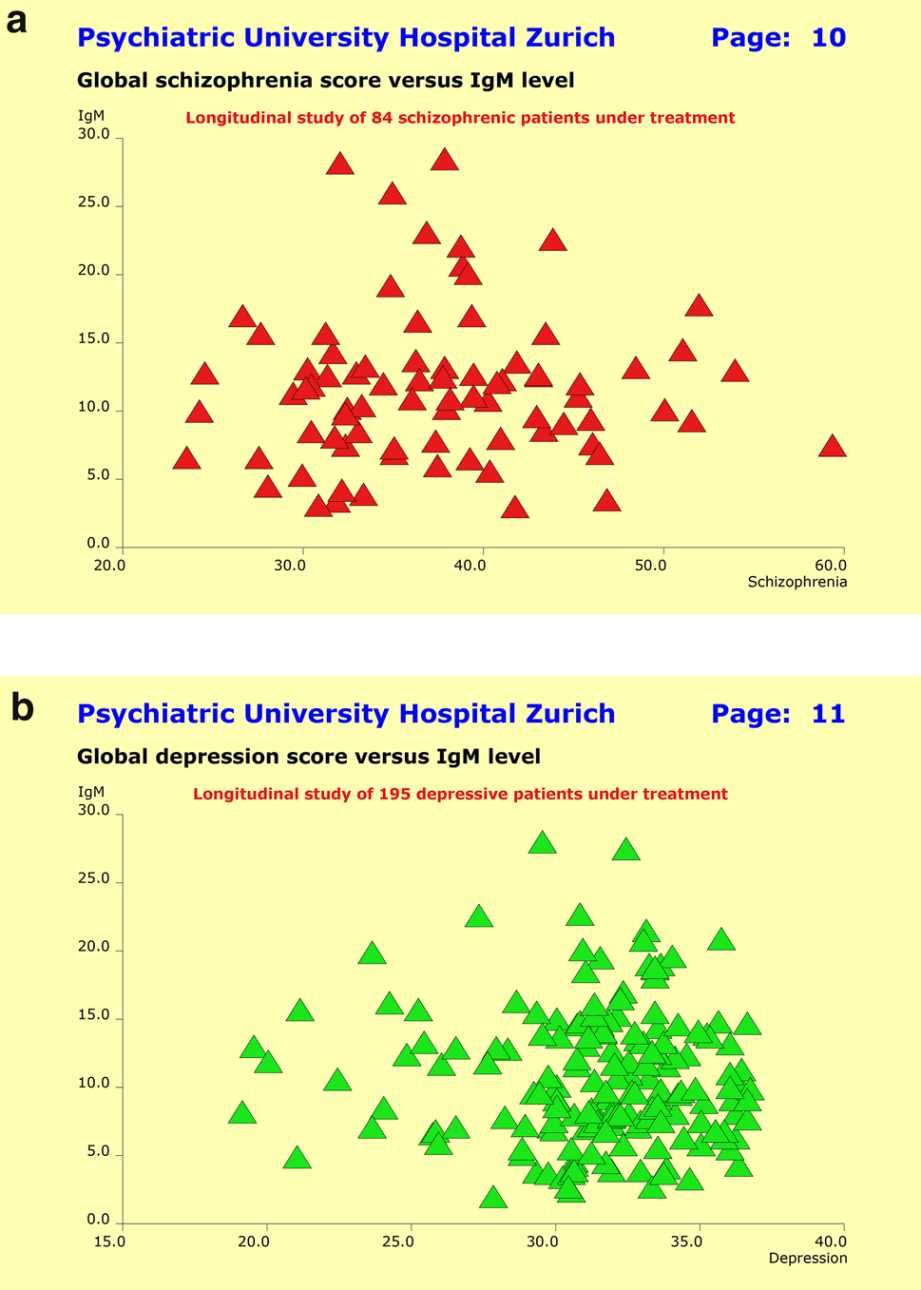
**a** Tentative analyses did not reveal any substantial association between clinical picture and treatment outcome on one hand, and IgM levels on the other. Neither for the parameters “diagnosis”, “previous history”, “severity at baseline”, “age”, “gender”, and “unwanted side effects” nor for the subgroups of treatment responders or non-responders. By contrast, the scatter plot “global schizophrenia score” (horizontal axis) versus IgM levels (vertical axis) suggested the existence of a subgroup of patients for whom the anti-inflammatory response system may have had influenced the patients’ clinical picture and response to treatment. **b** Tentative analyses did not reveal any substantial association between clinical picture and treatment outcome on one hand, and IgM levels on the other. Neither for the parameters “diagnosis”, “previous history”, “severity at baseline”, “age”, “gender”, and “unwanted side effects” nor for the subgroups of treatment responders or non-responders. By contrast, the scatter plot “global depression score” (horizontal axis) versus IgM levels (vertical axis) suggested the existence of a subgroup of patients for whom the anti-inflammatory response system may have had influenced the patients’ clinical picture and response to treatment

The previous history syndrome scores included the SSCL-16 scales “schizophrenic thought disorders”, “delusions”, “hallucinations”, “ego consciousness”, “anergia”, “incongruent affect”, and “depressive syndrome” while baseline severity was assessed through HAM-D21 and PANSS-G scores. Due to incomplete data, four F2 patients (4.8%) had to be excluded from the analysis, along with sixteen F3 patients (8.2%). In accordance with the primary design of this study, we iteratively optimized two independent models, one for the F2 patients and the second for the F3 patients. Up to 45 M. iterations were necessary to fit the models sufficiently well to the empirical data (Fig. 7).

**Fig. 8:**
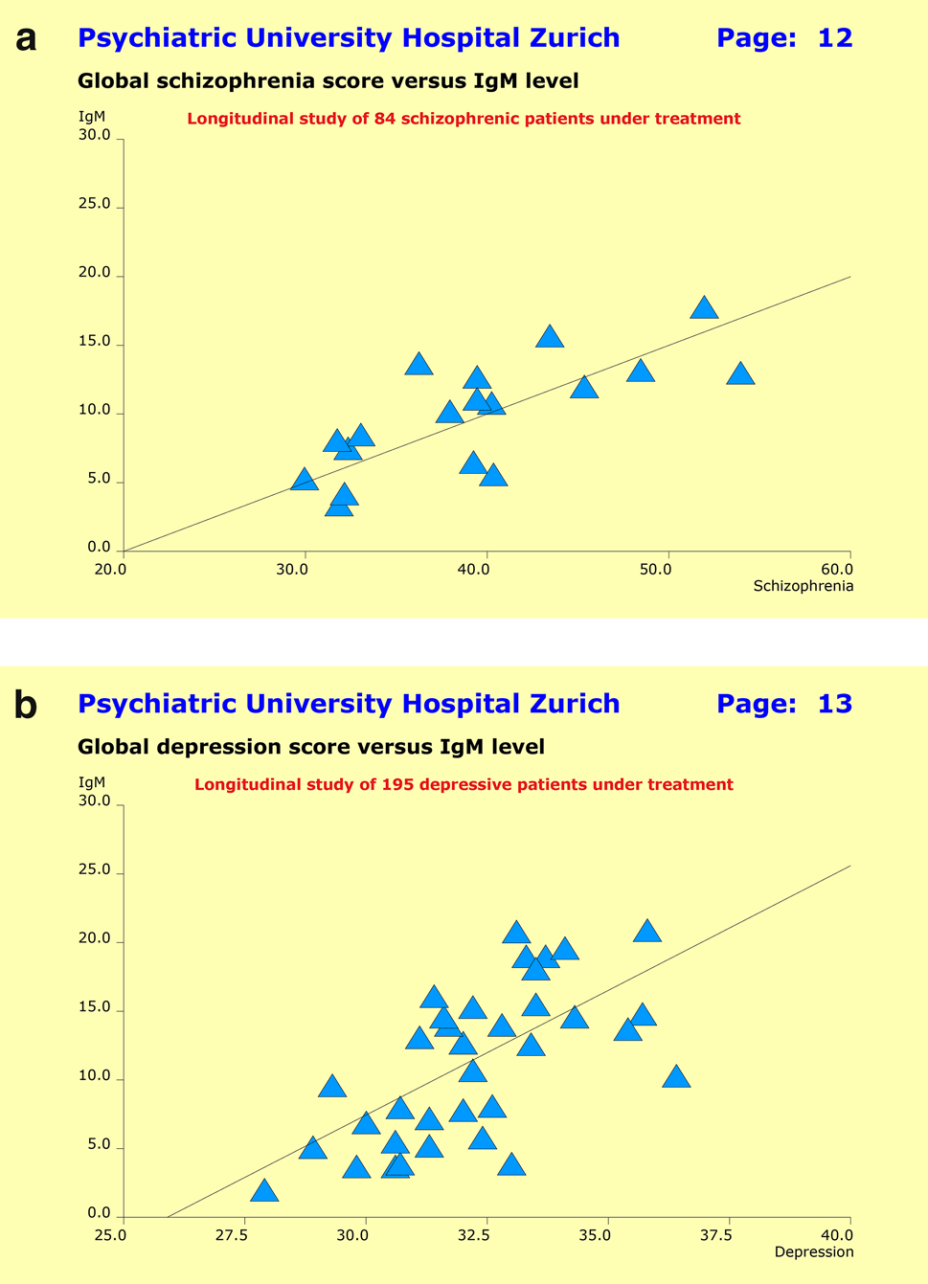
**a** For the F2 patients, our NN model identified a 22.5% subgroup exhibiting a significant correlation of *r* = 0.746 (*p* = 0.0004) between global schizophrenia scores and IgM levels, along with a correct prediction of response of 94.4%, thus explaining 55.7% of the observed between-patient variance. No simple IgM cutoff value applies. **b** For the F3 patients, our NN model identified a 19.6% subgroup exhibiting a significant correlation of *r* = 0.644 (*p* = 0.00003) between global depression scores and IgM levels, along a correct prediction of response of 89.6%, thus explaining 41.4% of the observed between-patient variance. No simple IgM cutoff value applies

For the patients with primary F2 diagnoses, the final NN model yielded a 22.5% subgroup (*n* = 18) with a highly significant linear correlation of *r* = 0.746 (*p* = 0.0004) between the global schizophrenia score and IgM levels, and a false-positive prediction error rate of 5.6%. The model explained 55.7% of the observed between-patient variance (Fig. 8a). By contrast, for the patients with primary F3 diagnoses the final NN model yielded a slightly smaller 19.6% subgroup (*n* = 35) with a similarly significant linear correlation of *r* = 0.644 (*p* = 0.00003) between the global depression score and IgM levels, and a false-positive prediction error rate of 11.4%. The model explained 41.4% of the observed between-patient variance (Fig. 8b). It is worth noting that Fig. 8a, b exclude simple IgM cutoff values.

The scatter plots of Fig. 7a, b did not exclude a negative correlation between the patients’ clinical pictures and IgM levels as well. Consequently, we have rerun the above two NN models, this time explicitly demanding negative correlations. As expected, these models could easily be fitted to the empirical data. The models’ predictive power—in terms of achievable false-positive error rates regarding treatment outcome—turned out to be rather unsatisfactory. We have therefore decided to retain the original models.

The patients in the overlap zone showed, on average, higher IgM levels compared to the rest of the sample, which in the case of the F2 patients reached statistical significance (*p* = 0.0425). Yet attempts failed so far to find a multi-layer NN model that “explained” sufficiently well the diagnostic overlap (dependent variable) in the plane spanned by the global “Depression” and “Schizophrenia” scales (cf. Fig. 3) through IgM levels in combination with other parameters (independent variables).

## Discussion

In this naturalistic study, an attempt was made to assess today’s acute inpatient treatment of major depressive and schizophrenic disorders as comprehensively and in detail as possible: common therapeutic strategies, medications, unwanted side effects, time course of recovery, and efficacy of treatments. A particular focus of attention was laid on (1) the efficacy of polypharmacy regimens in comparison to monotherapy; and (2) the potential involvement of inflammatory processes in the pathogenesis of depressive and schizophrenic disorders in an initially unknown subgroup of patients.

### Polypharmacy

Results showed that monotherapy and psychotherapy without supplemental psychotropic drugs have ceased to be the treatment options of choice in today's acute psychiatry. One rarely ever finds patients receiving less than two or more medications—even among mild cases. In other words, polypharmacy is ubiquitous in today’s acute treatment of depressive and schizophrenic disorders. No rational strategy was discernible in the diversity of polypharmacy regimens. The parameter “ward” (i.e., psychiatrist in charge) turned out to be the by far most determining factor, followed by gender, age, and diagnosis. Severity at baseline did not play any role.

While the share of polypharmacy in the acute treatment of psychiatric disorders is still increasing in the Western world, there seems to be a trend reversal in Asia. In a recent study that examined the trends in psychotropic polypharmacy over the years 2004–2013 in East Asia (China, Japan, Korea, Singapore, and Taiwan), the authors found significant decreases regarding the use of polypharmacy in the three diagnostic categories under investigation “mood disorders”, “anxiety disorders”, and “psychotic disorders” [6]. Yet at the same time, the proportion of patients with a diagnosis of mood disorders and receiving antipsychotics in combination with antidepressants showed a significant increase, as was the case for the proportion of patients with a diagnosis of psychotic disorders and receiving mood stabilizers in combination with antipsychotics [6].

Evidentially, the results of our present study relate to the acute treatment of patients with depressive or schizophrenic disorders. The situation may be somewhat different for the maintenance treatment of patients with schizophrenic disorders where the combination of antipsychotics can have beneficial effects as suggested by a recent nationwide Finish cohort study of 62,250 individuals with schizophrenia and up to 20 years follow-up [10]. But overall, the evidence of a more favorable long-term outcome in maintenance treatment under a combination of drugs when compared to monotherapy is scarce as pointed out by a comprehensive Cochrane review study [11]. Therefore, the use of polypharmacy in the maintenance treatment of schizophrenic disorders must necessarily remain an issue of controversy and discussion.

### Side effects

We found a close link between treatment regimen and unwanted side effects. The mere number of drugs taken simultaneously explained a highly significant (*p* < 0.001) proportion of the observed variance in side effects irrespective of the substances involved: 17.8% of the variance of sexuality-related side effects, 11.8% of the variance in gastrointestinal disturbances, 13.3% of the variance in cardiac-respiratory disturbances, and 18.4% of the variance in cardiovascular disturbances. For severe side effects, certain drugs can have a much stronger side effect pattern than others. A tentative nonlinear NN model of drugs along with their interactions could explain a major proportion of up to 50% of the observed variance in severe side effect patterns.

The most disturbing aspects of our results, however, are the findings that (1) the overall responder rates have dropped by 10–15% in the last 2 decades when compared to earlier double-blind monotherapy studies [e.g., [38]]; and (2) the over-proportionally high response rates among severely to very severely ill patients, as it used to be the norm some 15 years ago, are no longer observed any more [e.g., [39]]. The widespread pre-treatment of today’s patients with antidepressants and antipsychotics by the family doctor (one rarely ever sees drug-naïve patients any more) combined with polypharmacy have certainly contributed to a good deal to this unfavorable development [cf. 40,41].

### Inflammatory response system

The most important question investigated by our study concerned the amount of between-patient variance that was “explainable” by chronically elevated levels of the “natural” antibody immunoglobulin M (IgM). For the sample as a whole entity, our analyses yielded no significant association between IgM levels and psychopathology syndrome scores. However, it was readily possible to “construct” a 22.5% subgroup of patients (F2 diagnoses) and a 19.6% subgroup of patients (F3 diagnoses) for which highly significant correlations showed up between IgM levels on one hand, and the global schizophrenia and depression scores on the other. The NN models explained with 55.7% and 41.4%, a major proportion of the observed between-patient variance while predicting response to treatment at low false-positive error rates of 5.6% and 11.4%. False-negative prediction errors could not be reliably estimated since (1) other subgroups of patients are likely to exhibit treatment responders as well; and (2) the “true” response rates are unknown and the observed rates are likely biased by polypharmacy and pre-treatments.

The observed close link between IgM levels and psychopathology syndrome scores does not imply simple IgM cutoff values and must not necessarily be causal. Our findings could well be attributed to some unknown background process which influences the anti-inflammatory response system, thereby increasing the unspecific vulnerability to psychiatric disorders in general [42]. The results confirm our previous models [12, 43] and extend them by data regarding the time course of recovery under treatment and by NN-derived classifiers that can predict response to therapy with good certainty. These classifiers replicate similar reports in the literature where, for example, (1) inflammatory biomarkers served as a differential predictor of outcome of depression treatment with escitalopram and nortriptyline [24],or (2) low-grade inflammation predicted antidepressant and anti-inflammatory therapy response in MDD patients [25]. Therefore, the study has likely added another line of evidence to the complex puzzle of antidepressant and antipsychotic drug response, where non-steroidal anti-inflammatory drugs, acetylsalicyclic acid, COX-2 inhibitors showed significant positive effects as adjunctive treatments in major depression [26], and schizophrenic disorders [26, 32]. As to disentangling the etiologic heterogeneity underlying the clinical diagnoses of depressive and schizophrenic disorders, significant contributions come from (1) recent molecular-genetic studies regarding the FKBP5 gene expression, which appears to predict antidepressant treatment outcome in depression [44, 45], (2) the success of the combined dexamethasone/CRH test as a potential surrogate marker in depression [46, 47],and (3) the description of the potential link between microglial activation and progressive brain changes in schizophrenia [28].

In this context, one must necessarily also consider the factor "chronic stress", which not only plays an important role in the pathogenesis of psychiatric disorders [48–50], but can also significantly influence the efficacy of therapeutic interventions [51, 52]. Here come “early detection” and “prevention” into play. The method of approach to quantifying basic coping behavior proposed by Mohr et al. [48] and Zhang et al. [49], along with other risk factors, appears to be a promising way for such an “early” detection of subjects with an elevated risk of stress-related mental health problems, nota bene prior to the development of clinically relevant symptoms. The socio-economic impact of the potential prevention of depressive disorders, and psychiatric disorders in general, can be enormous.

Our results suggest the following implications for everyday clinical practice: (1) we have to accept that psychotropic drugs are ineffective or insufficiently effective in a larger proportion of patients; (2) polypharmacy does not solve this problem in any way—patients often have no benefits whatsoever, only the disadvantage of stronger side effects; (3) we have further to accept that psychiatric disorders, as they are visible through the clinical picture of patients, are likely the result of etiologically very different disease developments, i.e., psychiatric disorders do not represent disease entities in terms of prognosis and therapy; (4) in light of this, it is principally quite unlikely that all patients respond equally well to a particular therapy and, consequently, we have to also think about alternatives, such as the inflammatory response system, as targets for therapeutic intervention [cf. 53]; (5) F2 patients with a major depressive component are the most promising candidates in this respect; (6) in contrast to everyday clinical practice, monotherapy is by no means obsolete—empirical evidence speaks against starting just every therapeutic intervention in psychiatry with a combination of psychopharmaceuticals; (7) finally, for mild depression (HAM-D17 baseline score < 22), for example, we should consider to not use psychopharmaceuticals at all, or to opt for psychotherapy alone—therapeutic approaches that are worth to be checked carefully in each individual case.

### Limitations

According to the study design, all new hospital admissions were informed about the objectives of our study and invited to participate in this research project. Specifically, patients were informed that participation is voluntary and that they can cancel their participation at any time without giving reasons and without having any disadvantage. Due to the voluntary nature of our study, the obtained selection of study participants must not necessarily be representative of the targeted total population of F2 and F3 patients. For example, the chosen recruitment procedure may have resulted in a bias towards more cooperative patients, or towards milder cases, or in the opposite direction towards more severe cases. By design, we have no way to detect such selection biases.

Although of quite respectable size, our sample was not large enough to derive a sufficiently accurate estimate of the differences “polypharmacy versus monotherapy under acute treatment”. Nonetheless, we think that our findings regarding side effect patterns and overall efficacy of therapeutic interventions provide a sound basis to draw reliable conclusions for everyday clinical practice.

Even though we applied the *k*-fold cross-validation approach to avoid testing on the training data and over-optimistic classification rates, we cannot expect that the retrospectively constructed classifiers will work equally well in a prospective design. Independent learning and testing sample sets are required to achieve this goal.

## Conclusions

Polypharmacy is omnipresent in today’s acute treatment of depressive and schizophrenic disorders. Given the large proportion of patients with unwanted side effects and the strong correlation between side effects and the number of drugs, our results suggest that polypharmacy approaches are not equally suited for every patient. Moreover, polypharmacy appears to have a negative impact on treatment response and obfuscates the “natural” time course of recovery through a multitude of interfering factors. In terms of efficacy, there are no advantages of polypharmacy over monotherapy, a finding which underlines the need for more personalized treatment solutions in psychiatry. It is of major clinical relevance that our study appears to have cleared the way for the early identification of a subgroup of patients suffering from major psychiatric disorders for whom the inflammatory response system is a promising target of therapeutic intervention.

## Electronic supplementary material

Below is the link to the electronic supplementary material.

Supplementary file1 (PDF 251 kb)

Supplementary file2 (PDF 221 kb)

## Footnotes

1 Depressive disorders can be a risk factor for the development of rheumatoid arthritis [53].

2 IgM autoantibodies are proteins produced by the immune system in response to a perceived threat—which also may be the result of an aberrant immune response of the organism against its own cells and tissues (autoimmune inflammatory processes).

3 The PANSS G scale was chosen to prioritize illness-related disabilities in daily functioning over acute symptomatology.

4 We used the P scale as response criterion, since only this scale reflects the rapid response of patients to therapeutic interventions. This is in contrast to the N and G scales, where psychopathology is only slightly reduced, if at all, during the study period.

5 Comparisons were made both with this study sample (*n*=279) and with our previous data (*n*=2,788) that showed highly significant advantages of the monotherapeutic approach. The latter comparison, however, is somewhat unfair because response rates have generally fallen to a worrying degree over the past 15 years, so that data are not directly comparable. Results are therefore not included.
