# Supplementary material for: Inflammatory processes linked to major depression and schizophrenic disorders and the effects of polypharmacy in psychiatry: evidence from a longitudinal study of 279 patients under therapy
Source: Eur Arch Psychiatry Clin Neurosci. 2020 Jul 21;271(3):507–20. doi: 10.1007/s00406-020-01169-0 (PMC7981316; doi:10.1007/s00406-020-01169-0)
Supplement: Supplementary file 1 — Supplementary file1 (PDF 251 kb) [file 406_2020_1169_MOESM1_ESM.pdf]

Psychiatric University Hospital Zurich, Division of Clinical Psychiatry

MEDICATION AND SIDE EFFECTS

M E D I S

St. Kuny

**STUDY**	[_ _ _ _]	1-4
**GROUP**	[_ _]	5-6
**PATIENT**	[_ _ _]	7-9
**RATING DAY**	[_ _ _]	10-12
**CARD NUMBER**	[_ _]	13-14
Sex (1=male, 2=female)	[_]	15
Birthday (dd.mm.yyyy)	[_ _ : _ _ : _ _ _ _]	16-23

**DATE** (dd.mm.yyyy)	[_ _ : _ _ : _ _ _ _]	54-61
**INTERVIEWER**	[_ _ _]	62-64
**HOSPITAL**	[_ _]	65-66
**PATIENT ID** (the hospital's internal PID)	[_ _ _ _ _ _ _ _ _ _ _ _]	67-78

University of Zurich[UZH]
Institute for Response-Genetics

© 2015

Bitte zunächst nur Medikamenten<u>namen</u> und Dosis einsetzen. Der dreistellige <u>Code</u> der Medikamente (s. S. 4) wird später vom Studienadministrator nachgetragen.

1-12 dupl

Card number [_ _] 13-14

1 **1. Medikament**
Medikamentenname [______________________________] [_ _ _] 15-17
Dosis [_ _ _ _ . _] 18-22

2 **2. Medikament**
Medikamentenname [______________________________] [_ _ _] 23-25
Dosis [_ _ _ _ . _] 26-30

3 **3. Medikament**
Medikamentenname [______________________________] [_ _ _] 31-33
Dosis [_ _ _ _ . _] 34-38

4 **4. Medikament**
Medikamentenname [______________________________] [_ _ _] 39-41
Dosis [_ _ _ _ . _] 42-46

5 **5. Medikament**
Medikamentenname [______________________________] [_ _ _] 47-49
Dosis [_ _ _ _ . _] 50-54

6 **6. Medikament**
Medikamentenname [______________________________] [_ _ _] 55-57
Dosis [_ _ _ _ . _] 58-62

1-12 dupl

Card number [_ _] 13-14

7 **7. Medikament**
Medikamentenname [______________________________] [_ _ _] 15-17
Dosis [_ _ _ _ . _] 18-22

8 **8. Medikament**
Medikamentenname [______________________________] [_ _ _] 23-25
Dosis [_ _ _ _ . _] 26-30

9 **9. Medikament**
Medikamentenname [______________________________] [_ _ _] 31-33
Dosis [_ _ _ _ . _] 34-38

10 **10. Medikament**
Medikamentenname [______________________________] [_ _ _] 39-41
Dosis [_ _ _ _ . _] 42-46

11 **11. Medikament**
Medikamentenname [______________________________] [_ _ _] 47-49
Dosis [_ _ _ _ . _] 50-54

12 **12. Medikament**
Medikamentenname [______________________________] [_ _ _] 55-57
Dosis [_ _ _ _ . _] 58-62

13 **Körpergewicht und Blutdruck (bei jeder Untersuchung neu bestimmen)**

Körpergewicht [_ _ _ . _] 63-66
Blutdruck (systolisch/diastolisch) [_ _ _] 67-69
[_ _ _] 70-72

Nebenwirkungen

(0= nicht vorhanden, 1= fraglich vorhanden, 2= leicht, 3= mittel, 4= schwer, 5= sehr schwer)

1-12 dupl

0. Card number [_ _] 13-14

1. Schlaf- und Vigilanzstörungen

Einschlafstörungen	[_]	15
Durchschlafstörungen	[_]	16
Verkürzung der Schlafdauer	[_]	17
Früherwachen	[_]	18
Müdigkeit am Tag	[_]	19

2. Appetenzstörungen

Appetit vermindert	[_]	20
Appetit vermehrt	[_]	21
Durst vermehrt	[_]	22

3. Sexualität

Sexualität vermindert	[_]	23
Sexualität vermehrt	[_]	24
Sexueller Vollzug gestört	[_]	25

4. Gastro-intestinale Störungen

Hypersalivation	[_]	26
Mundtrockenheit	[_]	27
Übelkeit	[_]	28
Erbrechen	[_]	29
Magenbeschwerden	[_]	30
Obstipation	[_]	31
Diarrhoe	[_]	32

5. Kardio-respiratorische Störungen

Atembeschwerden	[_]	33
Schwindel	[_]	34
Herzklopfen	[_]	35
Herzdruck	[_]	36

6. Andere vegetative Störungen

Akkomodationsstörungen	[_]	37
Schwitzen vermehrt	[_]	38
Seborrhoe	[_]	39
Miktionsstörungen	[_]	40
Menstruationsstörungen	[_]	41

7. Weitere Störungen

Kopfdruck	[_]	42
Rückenbeschwerden	[_]	43
Schweregefühl in den Beinen	[_]	44
Hitzegefühl	[_]	45
Frösteln	[_]	46
Bewegungsstörungen	[_]	47

8. Neurologische Störungen

Rigor	[_]	48
Muskeltonus erniedrigt	[_]	49
Tremor	[_]	50
Dyskinesien	[_]	51
Hypokinesien	[_]	52
Akathisie	[_]	53
Ataxie	[_]	54
Nystagmus	[_]	55
Parästhesien	[_]	56

9. Kardiovaskuläre Störungen

Orthostatische Hypotension	[_]	57
Bluthochdruck	[_]	58
Rhythmusstörungen	[_]	59
Blutbildveränderungen	[_]	60

**Seitendifferenzen (0=nein, 1=ja)**	[_]	61
**Cerebrale Krampfanfälle (0=nein, 1=ja)**	[_]	62

001 Abilify 5 10 15 30 mg	049 Haldol 1 10 mg	097 Sinquan (Aponal, Doneurin) 50 mg
002 Akineton 2 mg	050 Haldol Decanoas 50 100 mg/ml	098 Solatran 15 30 45 mg
003 Akineton ret 4 mg	051 Hova 200/46 mg	099 Solian 100 200 400 mg
004 Akineton Ampullen 1 mg/ml	052 Imovane 7.5 mg	100 Sulfarlem S 25 25 mg
005 Anafranil 10 25 mg	053 Inderal 10 40 mg	101 Subutex 0.4 2 8 mg
006 Anafranil SR 75 mg	054 Insidon 50 mg	102 Surmontil (Herphonal, Stangyl) 25 100 mg
007 Aphenylbarbit 100 mg	055 Jarsin 300 mg	103 Tegretol CR 200 400 mg
008 Atarax 25 mg	056 Jatrosum N 10 mg	104 Temesta (Tavor) 1 2.5 mg
009 Aurorix 150 mg	057 Keppra 500 mg	105 Temesta expidet 1 2.5 mg
010 Becozym forte	058 Lamictal (Lamotrigin) 25 50 100 mg	106 Tenormin 50 100 mg
011 Beloc Zok 50 95 mg	059 Leponex 25 100 mg	107 Timonil retard 300 mg
012 Benerva 100 300 mg	060 Lexotanil 1.5 3 mg	108 Tofranil 10 25 mg
013 Benocten 50 mg	061 Lithiofor 660 mg	109 Tolvon 30 60 mg
014 Buspar 10 mg	062 Ludiomil 25 75 mg	110 Topamax 15 50 100 mg
015 Campral 300 mg	063 Lyrica 25 ...300 mg	111 Torecan 6.5 mg
016 Chloraldurat rot 250 mg	064 Magnesiocard 10 mmol	112 Tranxilium 5 10 20 50 mg
017 Cipralex 10 mg	065 Mefenacid 250 500 mg	113 Trilafon (Decentan) 4 8 mg
018 Clopixol (Ciatyl Z) 2 10 25 mg	066 Methadon streuli 5 mg	114 Trileptal 300 mg
019 Clopixol Tropfen 20 mg/ml	067 Mysoline 250 mg	115 Tript-OH 100 mg
020 Clopixol Decanoas 20 mg/ml	068 Modasomil 100 mg	116 Trittico (Thombran,Trazodon) 50 100 mg
021 Concerta retard 36 54 mg	069 Neurontin 400 mg	117 Truxal 15 50 mg
022 Concor 10 mg	070 Nexium 20 mg	118 Urbanyl 10 mg
023 Dafalgan 500 mg	071 Nortrilen 25 mg	119 Valium 2 5 10 mg
024 Dalmadorm 15 30 mg	072 Noveril TR 240 mg	120 Valverde Schlaf 250/60 mg
025 Dapotum 5 mg	073 Nozinan 25 100 mg	121 Xanax 0.25 0.5 1 mg
026 Dapotum D 25 100 mg/ml	074 Orfiril (Ergenyl,Leptilan) 300 500 1000 mg	122 Xanax retard 0.5 1 mg
027 Deanxit 0.5/10 mg	075 Priadel 400 mg	123 Zoloft 50 mg
028 Demetrin 10 20 mg	076 Prazine 25 50 mg	124 Zolpidem (Stilnox) 10 mg
029 Depakine chrono 300 500 mg	077 Psychopax 12.5 mg/ml	125 Zyprexa 1.5 5 10 15 mg
030 Depakine Lösung 300 mg/ml	078 Quilonorm 536 mg	126 Zyprexa Velotabs 5 10 15 20 mg
031 Deroxat 20 mg	079 Quilonorm retard 450 mg	127 Cipramil 20 40 mg
032 Dipiperon 40 mg	080 Remeron (Remergil) 30 45 mg	128 Cymbalta 30 60 mg
033 Distraneurin 192 mg	081 Risperdal 0.5 1 2 3 4 mg	129 Fluoxetin-Mepha 20 mg
034 Dogmatil 200 mg	082 Risperdal Lösung 1 mg/ml	130 Flusol 20 mg
035 Dormicum 7.5 15 mg	083 Risperdal Consta 25 37.5 mg	131 Gladem 50 100 mg
036 Edronax (Solvex) 4 mg	084 Ritalin Tbl 10 mg	132 Hyperval 250 500 mg
037 Efexor (Trevilor) 37.5 75 mg	085 Ritalin SR 20 mg	133 Paroxat 10 20 30 40 mg
038 Efexor ER (Trevilor ret.) 75 150 mg	086 Ritalin LA 20 30 40 mg	134 Paroxetin 20 mg
039 Eltroxin 0.1 0.05 mg	087 Rivotril Tbl 0.5 2 mg	135 Quilonum 450 mg
040 Entumin 40 mg	088 Rivotril Lös 0.1 mg/ml	136 Remotiv 250 mg
041 Eunerpan 5 mg/ml	089 Rohypnol 1 mg	137 Seralin-Mepha 50 100 mg
042 Floxyfral 100 mg	090 Saroten retard 25 50 mg	138 Stilnox CR 6.25 12.5 mg
043 Fluanxol 0.5 1 5 mg	091 Semap 20 mg	139 Tryptizol 10 25 75 mg
044 Fluanxol Decanoas 20 100 mg/ml	092 Serdolect 4 12 16 mg	140 Invega 3 6 9 12 mg
045 Fluctine 20 mg	093 Seresta 15 50 mg	141 Zeldox 20 40 60 80 mg
046 Fluctine 20 mg	094 Seropram 20 mg	142 Glianimon 5 10 mg
047 Gutron 2.5 mg	095 Seropram Tropfen 40 mg/ml	143 Taxilan 100 mg
048 Halcion 0.25 mg	096 Seroquel 25 100 200 300 mg	144 Wellbutrin (Elontril) 150 mg

145 Sonata 150 300 mg	193 Pantozol 20 40 mg	

146 Topamax 25 mg	194 Lescol 20 40 mg	
147 Sortis 10 20 40 mg	195 Valverde Schlaf forte 425 mg	
148 Zestril 5 10 20 30 mg	196 Andriol 40 80 120 mg	
149 Aspirin Cardio 100 300 mg	197 Symbicort 400	
150 Crestor 5 10 20 mg	198 MST Continus Retard	
151 Plavix 75 mg	199 Fluimucil 100 200 600 mg	
152 Tegretol 200 mg	200 Perenterol 50 250 mg	
153 Cosaar Plus 50 100 mg	201 Duphalac Sirup	
154 Seroquel prolong	202 Zocor 5 10 20 mg	
155 Glianimon 5 mg	203 Corotrend 5 10 20 mg	
156 Tremarit 15 mg	204 Simvastin	
157 Quilonum retard	205 Clamoxyl 500 mg	
158 Melneurin	206 Valdoxan 25 mg	
159 Taxilan 100 mg	207 Brufen 200 400 600 mg	
160 Valproat	208 Zylovic	
161 Lithium	209 Norvasc	
162 Tegretal ret.	210 Lamuna	
163 Equilibrin 30 60 90 120 mg	211 Prednisolon	
164 Dominal	212 Activelle	
165 Amitriptylin		
166 Stangyl		
167 Orap forte 4 mg		
168 Fluanxol Tropfen		
169 Metformin		
170 Panoral		
171 Calcimagon		
172 Spasmo Mucosolvan 30mg		
173 Folsan		
174 Ferro-Sanol duodenal 50mg		
175 Delix		
176 Delix 5 Plus		
177 Liviella		
178 Zestoretic		
179 Xyzal		
180 Coversum Combi 4 mg		
181 Isoptin 80 mg		
182 Vitamin B2 Streuli 5 10 mg		
183 Plendil Senior 2.5 mg		
184 Burgerstein Zink 15 mg		
185 Burgerstein Multivitamin		
186 Dilatrend 12.5 25 mg		
187 Glucophage 500 850 1000 mg		
188 Minirin 0.1 0.2 mg		
189 Fluomizin		
190 Voltaren 25 50 100 mg		
191 Adalat ret 20 40 60 80 mg		
192 Transipeg		
