# Supplementary material for: Inflammatory processes linked to major depression and schizophrenic disorders and the effects of polypharmacy in psychiatry: evidence from a longitudinal study of 279 patients under therapy
Source: Eur Arch Psychiatry Clin Neurosci. 2020 Jul 21;271(3):507–20. doi: 10.1007/s00406-020-01169-0 (PMC7981316; doi:10.1007/s00406-020-01169-0)
Supplement: Supplementary file 2 — Supplementary file2 (PDF 221 kb) [file 406_2020_1169_MOESM2_ESM.pdf]

Psychiatric University Hospital Zurich, Division of Clinical Psychiatry

SADS Syndrome Checklist

SSCL-16

Angst J., Scharfetter Ch., Stassen H.H. and Winokur G.

**STUDY**	[_ _ _ _]	1-4
**GROUP**	[_ _]	5-6
**PATIENT**	[_ _ _]	7-9
**RATING DAY**	[_ _ _]	10-12
**CARD NUMBER**	[_ _]	13-14
Sex (1=male, 2=female)	[_]	15
Birthday (dd.mm.yyyy)	[_ _ : _ _ : _ _ _ _]	16-23
Date of hospitalization (dd.mm.yyyy)	[_ _ : _ _ : _ _ _ _]	24-31
First diagnosis	[_ _ _ . _ _]	32-36
Second diagnosis	[_ _ _ . _ _]	37-41
Diagnostic system (1=ICD9, 2=ICD10, 3=DSM3-R, 4=DSM4)	[_]	42
Age at onset	[_ _]	43-44
Course (1=first manifestation, 2=intermittent, 3=progredient, 4=chronic)	[_]	45
Duration of Current Episode Prior to Hospitalization (days)	[_ _ _]	46-48
Medication Prior to Hospitalization (0=none, 1=antidepr., 2=neuroleptics, 3=other)	[_]	49
Current Medication (cf. list of codes)	[_ _ _]	50-52
Educational level (1=remedial, 2=junior high, 3=high, 4=college)	[_]	53
**DATE** (dd.mm.yyyy)	[_ _ : _ _ : _ _ _ _]	54-61
**INTERVIEWER**	[_ _ _]	62-64
**HOSPITAL**	[_ _]	65-66
**PATIENT ID** (the hospital's internal PID)	[_ _ _ _ _ _ _ _ _ _ _ _]	67-78

University of Zurich[UZH]
Institute for Response-Genetics

© 2012

0 = No information 1 = Absent 2 = Suspected or likely 3 = Definite

1-12 dupl

CARD NUMBER [_ _] 13-14

1. SCHIZOPHRENIC THOUGHT DISORDER

Schizophrenic thought disorder describes several readily identifiable, non-mutually exclusive forms of disturbance in speech or thinking. Disturbances in speech or thinking should not be counted as evidence of formal thought disorder if they are explainable by a transient overwhelming affect, (e.g., anger, fear), sarcasm, humor, limited intelligence or education, or language or cultural differences. Blocking, often thought of as indicative of formal thought disorder, is not included here because of the general unreliability of clinical judgments of this phenomenon, and because it is unlikely that it would be the only manifestation of formal thought disorder.

There is a tendency to overrate thought disorder when the subject is not definitely clear, concise, and logical. The rater should never rate any of these types of formal thought disorder unless he can give a specific example from the subject's speech.

Subject has had 1 or more distinct periods lasting at least 1 week (or any duration if hospitalized) with marked formal thought disorder, whether or not accompanied by blunted or inappropriate affect, delusions or hallucinations of any type, or grossly disorganized behavior. [_] 15

- Impaired understandability of speech due to psychopathology. This may be due to any combination of the following: lack of logical or meaningful connections between words, phrases, or sentences; excessive use of incomplete sentences which is not seen as a part of psychomotor retardation; excessive irrelevancies or abrupt changes in subject matter; distorted grammar or syntax; idiosyncratic word usage; use of empty, repetitious, stereotyped, or obscure phrases. [_] 16
- Loosening of associations (Derailment). Repeatedly saying things in juxtaposition which lack a readily understandable relationship, or shifting idiosyncratically from one frame of reference to another. [_] 17
- Illogical thinking (other than as evidenced by delusions, hallucinations, incoherence, loosening of associations, phobias or compulsions). Thinking in which facts are obscured, distorted or excluded. This is a complex and subtle judgment which usually requires a knowledge of the subject's reasoning process. [_] 18
- Poverty of content of speech. Speech is adequate in amount but conveys little information because of vagueness, talking past the point, empty repetitions or use of stereotyped or obscure phrases. Does not include poverty in the amount of speech. As Wing has noted, "This symptom may appear to be readily recognizable in some of one's colleagues, therefore only rate it when it is really pathological". [_] 19
- Neologism. Distortions of words, new words, uncommon combinations of words or fragments of words (contamination) invented by the subject, or standard words to which the subject has given new meaning. They may be invented to designate an experience which is beyond normal. [_] 20

**0 = No information**	**1 = Absent**	**2 = Suspected or likely**	**3 = Definite**

2. HYPOCHONDRIASIS

Has had 1 or more distinct periods lasting at least 1 week with excessive, inadequate concern with bodily functions or with real (but not harmful or dangerous) or imagined physical illness or disability. Rate the degree of preoccupation taking into account intensity, unreasonableness, and amount of time spent worrying. [_] 21

3. COMPULSION/PHOBIA

Has had 1 or more distinct periods lasting at least 1 week with obsessions or compulsions, which are defined as recurrent or persistent ideas, thoughts, images, feelings, impulses, movements or actions which generally are accompanied by a sense of subjective pressure and a desire to resist the event which is usually recognized by the individual as ego-alien. [_] 22

The obsessions or compulsions, or reactions to them, resulted in seeking or being referred for help from someone, taking medication, or impaired social functioning, within family, at home, or at work. [_] 23

Has had 1 or more distinct periods lasting at least 1 week of persistent and recurring irrational, inadequate fears of a specific object, activity, or situation which the subject tends to avoid. [_] 24

The phobic symptom(s), reactions to them, or behavior to avoid them, has resulted in either impairment in functioning, taking medication, or seeking or being referred for help from someone. [_] 25

4. DELUSIONS

Delusions are false personal convictions about the person himself and his world, which almost certainly are not true in the sense of inter-subjective reality and which are not shared with members of subject's religious or cultural subgroup. They may be fleeting in that the subject has a conviction in the truth of the belief for a brief time only, or they may be sustained. The subject's overt behavior may or may not be influenced by the delusional belief. A rating of "suspected" is appropriate when the rater suspects, but is not certain, that the subject has a particular delusion. Has had 1 or more distinct periods lasting at least 1 week with:

Delusions of being controlled (or influenced), or of thought broadcasting, insertion, or withdrawal. [_] 26

Delusions of reference. [_] 27

Persecutory delusions. [_] 28

Delusions of jealousy. [_] 29

Somatic, grandiose, religious, nihilistic, or other delusions without persecutory or jealous content. [_] 30

Extent to which the content of all of the delusions of any type (suspected or definite) is congruent with either an elated, irritable, or depressed mood. Frequently it is necessary to ask the subject the meaning of the delusions:

Unsystematic delusions, mood-congruent. [_] 31

Unsystematic delusions, mood-incongruent. [_] 32

0 = No information 1 = Absent 2 = Suspected or likely 3 = Definite

Systematic delusions, mood-congruent. [_] 33

Systematic delusions, mood-incongruent. [_] 34

5. HALLUCINATIONS

Hallucinations are perceptions, or perception-like experiences, in the absence of external stimulation. Hallucinations are recorded if they occurred when the subject was fully awake, and neither febrile nor under the influence of alcohol or drugs. Hallucinations should be confused neither with illusions, in which an external stimulus is misperceived, nor with exceptionally vivid normal thought processes. Always get the subject to describe the perception in detail. A rating of "suspected" indicates that the rater is not certain that the subject has experienced a hallucination, i.e., when it is not clear if the subject is describing an illusion rather than a true hallucination. If the hallucination occurred in the setting of a "religious experience", determine if this is an expected perception by the religious group the subject belongs to.

If the subject reports only unformed visual (spots, flashes, etc.) or auditory (roaring, ringing, rumbling, etc.) hallucinations, or only somatic or tactile hallucinations, the rater should be extremely cautious about scoring the symptom as definitely present. Such phenomena may be a drug reaction or an illusion rather than a hallucination. The item should be rated as "suspected" unless the rater is certain that it is a real hallucination.

Has had 1 or more distinct periods lasting at least 1 week with:

Auditory hallucinations in which either a voice makes a running commentary on the subject's behavior or thoughts as they occur, or 2 or more voices converse with each other. [_] 35

Visual hallucinations. [_] 36

Non-affective hallucinations of any type, the content of which is unrelated to depressed or elated mood (although subject may be depressed or elated at the time). Rate absent if limited to voices saying only 1 or 2 words. [_] 37

Extent to which the content of all of the hallucinations of any type (suspected or definite) is congruent with either an elated, irritable, or depressed mood. Frequently it is necessary to ask the subject the meaning of the hallucinations:

Auditory hallucinations, mood-congruent. [_] 38

Auditory hallucinations, mood-incongruent. [_] 39

Visual hallucinations, mood-congruent. [_] 40

Visual hallucinations, mood-incongruent. [_] 41

Other hallucinations, mood-congruent. [_] 42

Other hallucinations, mood-incongruent. [_] 43

0 = No information **1 = Absent** **2 = Suspected or likely** **3 = Definite**

6. DISORDER OF EGO CONSCIOUSNESS

Ego-consciousness is the certainty with which an individual is able to say "I am myself". Rate experience with diminution or loss of normal self-awareness concerning thoughts, feelings, actions, identity, body (e.g. uncertainty of being oneself, feelings of strangeness or of having changed). [_] 44

Thought broadcasting. Belief or experience that thoughts are broadcast from subject's head so that others could hear them. This may occur as a delusion and/or hallucination. The subject may hear his own thoughts from outside or within his head. Example: A man believed that his thoughts were being picked up by a hidden microphone and broadcast on TV. [_] 45

Thought insertion. The experience that thoughts, which are not one's own, are inserted into one's mind. This symptom is often falsely rated as present because it is confused with the belief that the subject has been caused to have unusual thoughts (for example, evil thoughts caused by the Devil), which are, nevertheless, his own. Do not include elated subjects who may speak as if their thoughts are coming from God (in such cases the subject may know they are his thoughts). Example: A man believed that thoughts were being put into his mind by radar. [_] 46

Odd or bizarre ideation or magical thinking, e.g., superstitions, clairvoyance, telepathy, "sixth sense", "others can feel my feelings", or suspected delusions in as far as they may be interpreted as a result of "ego-grandiosity" and/or defects in the "ego-boundary". [_] 47

Depersonalization or derealization. Feelings of being outside of one's body, or as if a body part does not belong to oneself, or as if one is physically cut off from other people, or as if one is floating, or that things seem unreal or unusually distant or veiled. Subject is neither febrile, nor under the influence of alcohol or drugs. [_] 48

Disturbances of "body-ego". Delusional belief that one's appearance, or an organ system, is diseased or changed. Examples: Brain is rotting, blood turning to ice. Do not rate mere excessive somatic concern. [_] 49

7. CATATONIA

Catatonic motor behavior: These symptoms are now rare and should only be considered present when they are obvious and have been directly observed by the rater or some other professional.

Catatonic stupor. Marked decrease in reactivity to the environment and reduction of spontaneous movements and activity. The patient may appear to be unaware of his surroundings. [_] 50

Catatonic rigidity. Patient maintains rigid posture against efforts to move him. [_] 51

Waxy flexibility. Patient maintains, for at least 15 seconds, postures into which he is placed. [_] 52

Catatonic excitement. Apparently non-intentional, sometimes stereotyped excited motor activity; not influenced by external stimuli. [_] 53

Catatonic posturing. Spontaneous assumption of inappropriate or bizarre posture. [_] 54

Bizarre behavior. Rate absent if due to simple depressive or manic syndrome, or to alcohol, drugs or impairment in brain functioning. [_] 55

0 = No information **1 = Absent** **2 = Suspected or likely** **3 = Definite**

8. ANERGIA

Subjective feeling of lack of energy or fatigue. (Do not confuse with lack of interest.) Rate as present even if clearly due to medication. [_] 56

9. INHIBITED-STUPOROUS

Retarded thinking and/or reduced spontaneous motor activity lasting at least two days. (Not catatonia and not due to drugs; includes psychomotor retardation and prolonged latency of response). [_] 57

10. AGITATED-EXCITED

Agitation lasting at least two days (not associated with manic syndrome). Includes inability to sit still, pacing, fidgeting, movements of lips or fingers, wringing hands, pulling at clothes. Not limited to isolated periods when discussing something upsetting. Do not include subjective feeling of tension or restlessness. [_] 58

Unusually energetic (lasting at least two days), more active than usual without expected fatigue. [_] 59

Less need for sleep than usual to feel rested (two or more days when subject needed less sleep). [_] 60

11. INCONGRUENT AFFECT

Inappropriate affect. Affect is incongruous with content of speech. For example, subject giggles while discussing reason for hospitalization. Do not include embarrassment or excessively strong affect, as when subject cries discussing a disappointment. [_] 61

Inadequate rapport (odd communication) in face-to-face interaction with constricted or inappropriate affect, e.g., aloof, distant, cold, superficial, silly. [_] 62

12. DEPRESSIVE SYNDROME

Has had 1 or more distinct periods lasting at least 1 week or, in the case of recurrent brief depression, frequent 2 — 3 day periods, during which subject has had depressive or irritable mood or has had pervasive loss of interest or pleasure. [_] 63

Sought or was referred for help from someone during dysphoric periods(s), took medication, or had impaired functioning socially, with family, at home, at work, or at school. [_] 64

During the most severe period he/she was bothered by:

- poor appetite or weight loss, or increased appetite or weight gain. [_] 65
- trouble sleeping or sleeping too much. [_] 66
- loss of energy, easily fatigued, or feeling tired. [_] 67

0 = No information **1 = Absent** **2 = Suspected or likely** **3 = Definite**

1-12 dupl

CARD NUMBER [_ _] 13-14

- loss of interest or pleasure in his/her usual activities or in sex (may or may not be pervasive). [_] 15
- feeling guilty or down on himself/herself. [_] 16
- trouble concentrating, thinking, or making decisions. [_] 17
- thinking about death or suicide (Did he/she attempt suicide?). [_] 18
- unable to sit still and have to keep moving. [_] 19
- feeling slowed down and having trouble moving. [_] 20

Had at least 3 (if past episode) or 4 (if current episode) of the above symptoms associated with the most severe period of depressed or irritable mood or pervasive loss of interest or pleasure. [_] 21

13. MANIC SYNDROME

Has had 1 or more distinct periods lasting at least 1 week (or any duration if hospitalized) when the predominant mood was either elevated, (i.e., unusually good, cheerful, high, expansive), or irritable (i.e., easily annoyed). (Do not include if apparently due to alcohol or drug use). Note: Frequently this item is falsely rated as positive when the subject is merely describing feeling very good in contrast to periods of depression. Don't forget to rate the hypomanic syndrome. [_] 22

Had at least 2 symptoms associated with the most extreme period of euphoric or irritable mood. (Do not include if due to alcohol or drug use.) [_] 23

During the most extreme period:

- was more active than usual – either socially, at work, at home, sexually, or physically. [_] 24
- was more talkative than usual or felt a pressure to keep on talking. [_] 25
- his/her thoughts raced or he/she talked so fast that it was difficult for people to follow. [_] 26
- felt he/she was a very important person, had special powers, plans, talents, or abilities (grandiosity). [_] 27
- needed less sleep than usual. [_] 28
- had trouble concentrating because attention kept jumping to unimportant things (distractibility). [_] 29
- has done something foolish or reckless that could have gotten him/her into trouble – like excessive spending, unwise business investments, sexual indiscretions or reckless driving. [_] 30

Symptoms were so severe that meaningful conversation was impossible, or there was serious impairment in functioning, or subject was hospitalized. [_] 31

0 = No information 1 = Absent 2 = Suspected or likely 3 = Definite

14. ATTEMPTED SUICIDE

Suicidal tendencies, including preoccupation with thoughts of death or suicide. (Do not include fears of dying.) [_] 32

Suicide Attempt: Seriousness judged by likelihood of being rescued, precautions against discovery, action to gain help during or after attempt, degree of planning, and the apparent purpose of attempt (manipulative versus killing oneself). [_] 33

0. No information or not sure.
1. Obviously no intent, purely manipulative gesture.
2. Not sure or only minimal intent.
3. Definite but very ambivalent.
4. Serious.
5. Very serious.
6. Extreme (careful planning and every expectation of death).

Medical lethality. Actual medical threat to life or physical condition following the most serious suicide attempt, taking into account the method (gunshot wound more serious than knife wound), impaired consciousness at time of rescue, seriousness of lesion, toxicity of ingested materials, reversibility (amount of time expected for complete recovery), and treatment required. [_] 34

0. No information or not sure.
1. No danger, e.g., no effects, held pills in hand.
2. Minimal, e.g., scratch on wrist
3. Mild, e.g., took 10 aspirins, mild gastritis.
4. Moderate, e.g., took 10 Seconals, briefly unconscious.
5. Severe, e.g., cut throat.
6. Extreme, e.g., respiratory arrest or prolonged coma.

Was unable to work (go to school, take care of the house), feed him/herself, dress him/herself, or keep him/herself clean because of suicidal attempt. [_] 35

15. AMNESTIC PSYCHOSYNDROME

Reduction/loss of memory or disorientation in time, place, or person. Often in combination with emotional lability and intellectual impairment. [_] 36

16. OTHER SYMPTOMS

Psychiatric conditions which cannot be classified in any of the previous categories. [_] 37

The clinical picture suggests a specific disorder not covered by this instrument. [_] 38

Etiology is most likely to be known organic factor. [_] 39

17. HEIGHT / WEIGHT

Height [cm] [_ _ _] 40-42

Weight [kg] [_ _ _ . _] 43-46
